# Continuous, non-invasive measurement of the haemodynamic response to submaximal exercise in patients with diabetes mellitus: evidence of impaired cardiac reserve and peripheral vascular response

**DOI:** 10.1136/hrt.2009.177113

**Published:** 2009-10-22

**Authors:** D Joshi, A Shiwalkar, M R Cross, S K Sharma, A Vachhani, C Dutt

**Affiliations:** 1 Torrent Research Centre, Village Bhat, Gandhinagar, Gujarat, India; 2 Veeda Clinical Research, Old Convent of Notre Dame, Derriford, Plymouth, UK; 3 Veeda Clinical Research, Ambawadi Ahmedabad, Gujarat, India

## Abstract

**Background:**

Reduced exercise capacity in diabetics has been attributed to limitations in cardiac function and microvascular dysfunction leading to impaired oxygen supply and nutritive perfusion to exercising muscles.

**Objective:**

To study changes in cardiac function and microvascular utilisation during exercise in diabetic individuals compared to age-matched controls.

**Methods:**

Diabetics with glycosylated haemoglobin (HbA_1c_) <8 (n = 31), diabetics with HbA_1c_ ⩾8 (n = 38) and age-matched non-diabetic controls (n = 32) performed exercise at 50 W for 10 minutes followed by recovery, with continuous monitoring of cardiac function by impedance cardiography and regional flow and oxygen saturation by laser Doppler and white light spectroscopy.

**Results:**

In the diabetics, cardiac reserve during exercise and cardiac overshoot during recovery are significantly reduced because of reduction in capacity to increase stroke volume. Regional flow to the exercising muscle is reduced and there is also disproportionately greater desaturation of the regional flow. Abnormalities in cardiac function and regional perfusion are related to the severity of diabetes.

**Conclusion:**

Cardiac response to exercise is attenuated significantly in diabetic individuals. Simultaneously, there is impairment in the regional distribution. These changes could be the harbinger of reduced exercise capacity in diabetics.

Clinical studies have shown that limitation in exercise capacity is a strong predictor of cardiovascular and all-cause mortality in diabetic patients with heart failure. Underlying pathophysiology is multifactorial and involves alterations in ventricular-vascular coupling consisting of cardiac changes on the one hand and compromised distribution at the regional microvasculature on the other. If such changes can be detected early (stage A) in those who are at risk of progressing to heart failure, it may help slow down progression by more aggressive management of the problem.

Though there is fair body of evidence on the mechanism underlying the decline in exercise tolerance and despite the recognition of its prognostic significance, monitoring microvascular changes underlying altered skeletal muscle exercise capacity and mapping them dynamically against cardiac response capacity has not been attempted. Monitoring of regional alterations in the microcirculation in patients is restricted by the invasive character of methodology and limitations to its adaptation to record continuous and dynamic change in the exercising muscle. Assessing cardiac function during exercise poses a special challenge as conventional echocardiographic measures including strain analysis, can only be done at rest or at the end of exercise. Therefore dynamic response to exercise, which is a function of continuous adjustment of pre-load (Frank-Starling) and after-load as well as direct cardiac adrenergic influence, designed to optimise the inotropic and chronotropic responses, cannot be captured by these methods.

Impedance cardiography (ICG) has been validated to gather ambulatory physiological recordings and was adapted to monitor cardiac function online during exercise.^1^ ^2^ For monitoring regional microcirculation this was combined with another method where laser Doppler shift caused by the movement of erythrocytes in the detected laser light is used to determine arteriolar flow. With the same probe, white light is introduced into the tissue and oxygen saturation of haemoglobin was determined from light absorbed by the haemoglobin in mixed capillary venous blood at a predetermined depth from the skin.^3^ This study was therefore aimed at investigating the sequential changes during mild exercise and recovery in cardiac function and regional microcirculation of exercising muscle in diabetes by a method which allows continuous dynamic monitoring, in order to capture early cardiac and vascular correlates, which could ultimately progress to impaired exercise tolerance. Submaximal exercise is more relevant as it is equivalent to the 6-minute walk test, used to evaluate exercise capacity in patients with marked left ventricular (LV) dysfunction.^4^ In contrast, maximum oxygen consumption (VO_2_max) may be compromised because of reduced patient motivation, poor peripheral blood flow and impaired skeletal muscle metabolism with early development of acidosis, besides limitation in cardiac output, correlates only modestly with this test.^5^

## Study protocol

### Subjects

The investigation conforms to the principles outlined in the Declaration of Helsinki.^6^ The study was approved by the institutional review board of Torrent Research Centre and Veeda Clinical Research (Ahmedabad, India). After signing the written informed consent, 127 subjects were screened. The inclusion criteria were subjects with confirmed diabetes as ascertained by history, clinical examination and laboratory investigations, who had no history of breathlessness on exertion or limitation in exercise capacity, and age-matched controls who were non-diabetic. Subjects with arrhythmia, significant peripheral vascular disease, retinopathy, insulin dependence and those with blood pressure (BP) >180/100 mm Hg, were excluded from the study. One-hundred and one subjects were included in the study and all completed the exercise protocol. Data from diabetics with glycosylated haemoglobin (HbA_1c_) <8 (n = 31), diabetics with HbA_1c_ ⩾8 (n = 38) and age-matched normal controls (n = 32) were included in the analysis.

### Experimental procedure

At Veeda Clinical Research, after their breakfast subjects had a brief physical examination, blood samples were collected for estimation of HbA_1c_, vascular cell adhesion molecule (VCAM), N-terminal brain natriuretic propeptide (NTProBNP) and urine samples for creatinine and microalbumin estimation. Oxygen to See (O2C, LEA Medizintechnik, Germany) probe LF3 incorporating Doppler (830 nm) and near infra-red light spectrum (500–850 nm), which allows measurement at skeletal muscle post-capillary venular site, was placed on the belly of the gastrocnemious on one leg. ICG (Bioz, Cardiodynamics, USA) and electrocardiogram (ECG-Mac5000, GE Healthcare, Germany) probes were placed on the chest and BP cuff on the arm.

After calibration, continuous measurements were obtained at baseline in the resting supine position for 10 minutes followed by the upright position for 10 minutes and then acclimatisation on the bicycle ergometer (eBike, GE Healthcare, Germany) for 10 minutes. Exercise was for a duration of 10 minutes, at a constant load of 50 W, on the electrically braked ergometer, varying rpm to maintain the constant load, which is equivalent to 4–7 METS. This level of exercise was chosen to simulate a 6-minute walk test commonly used to evaluate patients with heart failure in order to determine if it was adequate to evoke peripheral vascular response to differentiate the diabetic from non-diabetic subjects. At the end of the exercise period, subjects were asked to lie supine again for a recovery period of at least 15 minutes. ECG was recorded throughout and BP every 2 minutes. The same protocol was repeated in 10 subjects to assess the repeatability of the measurements. The entire study was completed over a period of four months.

### Data acquisition and processing

Data from O2C and ICG were acquired for five different phases—namely, resting supine baseline, standing, sitting on ergometer, during exercise and recovery after exercise. ICG measurements (collected every 1 minute) and O2C measurements (collected every 2 seconds were also averaged for 1 minute) were classified and synchronised for different phases of the protocol. Data were cleaned for machine error and artefacts (random and during transition from one phase of protocol to next) for all the measured variables. For O2C parameters, flow response to exercise was measured as area under the curve (AUC) and oxygen saturation (SO_2_) as area over the curve (AOC) of percentage change at each time point during exercise, from a mean of sitting. In addition, the slope to achieve maximum velocity in the microcirculation was computed. ICG parameters considered for analysis were cardiac output (CO), stroke volume (SV) and systolic time interval. Systolic time interval was obtained by summing the pre-ejection period (PEP) and left ventricular ejection time (LVET) obtained from ICG. Response during recovery was measured as AUC of the data points recorded during the recovery phase. Subjects who completed the exercise phase of 10 minutes were included for analysis on exercise data (table 1). For analysis on recovery data, only those who completed exercise followed by 15 minutes of recovery were included (28 diabetics with HbA_1c_<8, 35 diabetics with HbA_1c_ ⩾8 and 32 age-matched normal controls). To calculate oxygen debt for each subject, the value of mean SO_2_ at resting supine baseline, multiplied by the same period of time—that is, 15 minutes, was subtracted from AUC over 15 minutes of recovery.^7^ AUC has been calculated using WinNonlin (Version-5.2).

**Table 1:** Demographic profile of subjects

	Normal (n = 32)	HbA_1C_ <8 (n = 31)	HbA_1C_ ⩾8 (n = 38)
Age (years)	48.8 (5.3)	50.6 (7.3)	50.3 (4.6)
BMI (kg/m^2^)‡	23.81* (3.4)	26.42 (2.8)	26.57 (3.8)
Systolic blood pressure (mm Hg)	116 (10)	120 (12)	117 (11)
Diastolic blood pressure (mm Hg)	80 (9)	80 (7)	78 (9)
Cardiac output (l/ml)	4.96 (0.95)	5.10 (0.91)	5.19 (0.76)
Heart rate	77 (11)	80 (14)	80 (10)
NT-proBNP (pg/ml)†	31 (14.5–51.5)	35.1 (11.5–62.7)	27.6 (14.6–55.7)
VCAM (ng/ml)†	326.8 (186–647)	343.8 (171–617)	416.9 (253–611)
Diabetes duration (years)†	–	4 (3–10)	5 (3–10)
Microalbuminuria (mg/l)	18.99 (44.93)	17.85 (36.35)	41.05 (88.11)
eGFR (ml/min/1.73 m^2^)	85 (12)	80 (16)	90 (17)
Concomitant medication			
Secretagogues	–	70	73
Sensitisers	–	73	76
β-blockers‡	–	33	5**
Antihypertensives	–	50	34
Diuretics	–	7	3
Statins	–	8.3	0

Values are means (SD) or †median (interquartile range).

*p<0.01 normal vs <8, normal vs ⩾8; **p<0.05 <8 vs ⩾8.

‡Does not alter and contribute to diabetic vs non-diabetic comparison for various ICG and O2C variables.

### Statistical analysis

Data are expressed as mean (SD) and graphically represented as mean (SEM). Statistical analysis has been performed using SAS (Version-9.1). Demographic data have been tested to see differences among three groups using ANOVA followed by post-hoc Dunnett t tests (two-sided). Repeatability was determined by a two-sided paired t test on AUC values (derived from percentage change during exercise from sitting baseline) of the O2C parameters, SO_2_ and flow and ICG parameter, CO. Proportions of concomitant medications were subjected to Fisher’s exact test. CO, SV, time for downward inflection, and AUC or AOC of percentage change from baseline, during exercise and for recovery period, was compared between groups by the one-sided unpaired t test. In the absence of normality, data were subjected to the non-parametric Wilcoxon test. Percentage change in systolic time interval over time, of normal, diabetics with HbA_1c_ <8 and diabetics with HbA_1c_ ⩾8, were compared by repeated measures analysis of variance (RMANOVA). Statistical significance was defined as p value ⩽0.05.

## Results

### Demography

The demographic profile of the subjects included in the study is shown in table 1. Medications, sulphonylurea secretagogues, thiazolidinedione sensitisers, antihypertensives, angiotensin-converting enzyme (ACE) inhibitors and angiotensin receptor blockers (ARBs) and loop diuretics, as well as statins and β-blockers (table 1), taken by the subjects did not influence the measurements made during the study. Statins were analysed separately and did not show an effect on the cardiac response to exercise when comparing the two diabetic groups. Similarly, the diabetic group with HbA_1c_ ⩾8, showed a greater attenuation of the cardiac response to exercise as compared to the diabetic group with HbA_1c _<8. This was probably not secondary to attenuation of the response due to β-blockers as fewer diabetics with HbA_1c_ ⩾8 were taking β-blockers compared to those with HbA_1c_ <8.

### Cardiac response during exercise

**Figure 1:**
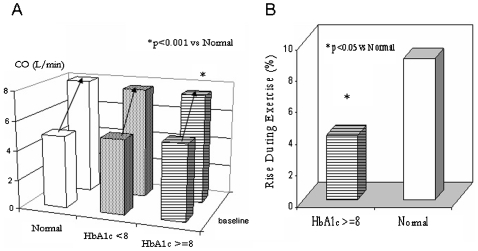
(A) Cardiac reserve during exercise and (B) stroke volume reserve during exercise, the key parameter limiting increase in cardiac output. CO, cardiac output; HbA_1c_, glycosylated haemoglobin.

**Figure 2:**
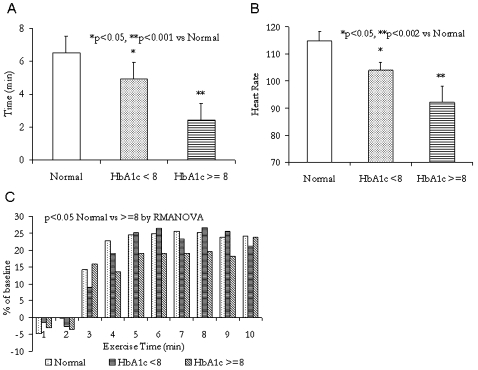
(A) Time to downward inflection of stroke volume response during exercise demonstrating that with increasingly poor glycaemic control there is increasing limitation in the capacity to augment stroke volume. (B) Heart rate at downward inflection in stroke volume during exercise. Increase in cardiac output occurs as a function of increase in heart rate with decreasing oxygen pulse delivered to muscles. (C) Reduction in systolic time interval compared to baseline indicating longer systole and compromised diastole in diabetics. HbA_1c_, glycosylated haemoglobin; RMANOVA, repeated measures analysis of variance.

Resting CO was lowest in normal individuals and although statistically not significant, it was slightly higher in the diabetic groups with HbA_1c_ <8 and ⩾8. In response to exercise, there was a rise in both SV and CO in all groups. However, the capacity to maximally increase CO in response to exercise, a measure of the cardiac reserve, was significantly (p<0.001) reduced in the diabetic patients compared to normal subjects (fig 1A). This could be ascribed to a smaller rise in SV during exercise in diabetic patients, which was significantly reduced (p<0.002) in the more severely diabetic group (fig 1B). Following the initial rise, the time to downward inflection towards reduction of SV, an indicator of inadequate filling secondary to increasing heart rate, occurred significantly (p<0.05 normal vs <8; p<0.001 normal vs ⩾8) earlier, and at lower heart rates (p<0.05 normal vs diabetic groups) in the diabetic groups compared to normal (fig 2A and 2B). Lengthening of the systolic time interval was observed in the diabetic group with HbA_1c_ ⩾8, in that there was a greater compensatory lengthening of the systolic time interval depicted here as a smaller fall in the systolic time for a given stroke output during exercise compared to normal (p<0.05) (fig 2C).

### Cardiac response during recovery

**Figure 3:**
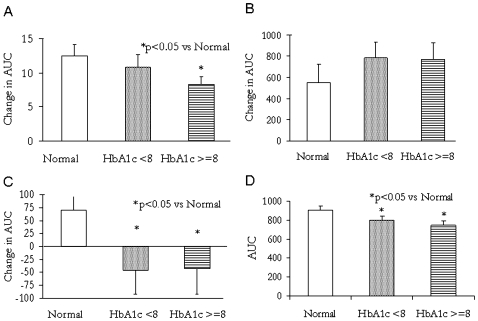
(A) Difference in area under the curve (AUC) of cardiac output (CO) during recovery from resting baseline: cardiac output overshoot during recovery. (B) Increase in regional flow over baseline during recovery: difference in AUC during recovery from resting baseline. (C) Oxygen debt: difference in AUC during recovery from resting baseline. (D) Oxygen saturation (AUC) in muscle flow during recovery. HbA_1c_, glycosylated haemoglobin.

During the recovery phase after exercise, the CO remained elevated and did not return to resting baseline. The increase however was attenuated in diabetics, more so in the diabetics with HbA_1c_ ⩾8 (p<0.05) (fig 3A). There was no difference in the heart rate response during recovery between the different groups.

### Skeletal muscle perfusion during exercise

**Figure 4:**
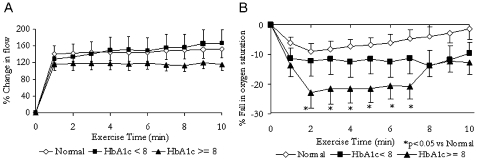
(A) Regional muscle flow during exercise and (B) oxygen saturation in blood supplied to exercising muscle. Note that in diabetics with reasonable control increase in flow is not able to contain desaturation. Diabetics with poor control show limited capacity to augment flow and run up a steeper oxygen debt with a late attempt at correction which could be due to local factors over-riding systemic vascular tone. HbA_1c_, glycosylated haemoglobin.

Regional changes in perfusion in the muscle as revealed by local measurement of flow are shown in figure 4A. There was a significant (p<0.05) reduction in AUC of percentage change from baseline in diabetic individuals with HbA_1c_ ⩾8 (1024.7 (780.3)) compared to normal subjects (1433.4 (1055.9)). Diabetics with HbA_1c_ <8, on the other hand, did not show a significant difference; however, to compensate, flow was slightly increased (1541.1 (1648.1)) compared to normal (fig 4A). This was accompanied by a reduction in oxygen saturation in both the diabetic groups compared to normal (fig 4B). AOC in normal subjects (−77.2 (170.8)) was significantly (p<0.05) more compared to diabetics with HbA_1c_ <8 (−125.3 (236.1)) and diabetics with HbA_1c_ ⩾8 (−165.6 (247.6)). This was offset, in the diabetics with HbA_1c_ ⩾8, by increased velocity of blood in the microcirculation, which showed a significant increase (p<0.05) in the slope to achieve maximal velocity (slope = 3.05 (2.59)), compared to the normal subjects (slope = 2.08 (1.65)).

### Skeletal muscle perfusion during recovery

Regional flow to the exercising muscle normally remains elevated, over resting levels, even after stopping exercise, during recovery. Compared to normal subjects, in diabetics augmentation of flow was more (fig 3B), in order to compensate for the reduced CO response during recovery.

Oxygen debt as determined from the diminished SO_2_ during recovery, over the resting baseline, was significantly increased in the diabetic subjects compared to normal (fig 3C) (p<0.05). Despite the increase in flow, desaturation of the local flow over the resting values persisted; however, it was least in the normal subjects, as evident by highest AUC of oxygen saturation during recovery. In the diabetic group, SO_2_ reduced significantly in both mild and severely diabetic patients (fig 3D).

### Repeatability

Data from 10 subjects who repeated the exercise protocol showed that AUC of percentage change from the sitting position to exercise did not show any significant difference in SO_2_ (507 (158) initial vs 625 (126) repeat, p>0.05), flow (3400 (867) initial vs 3166 (996) repeat, p>0.05) and in CO (75.6 (13.3) initial vs 75.7 (13.8) repeat, p>0.05). Hence, the precision of the relative measurements before and during exercise were reasonably accurate for acceptance of O2C and ICG.

## Discussion

This study characterises the haemodynamic response to exercise in diabetic individuals compared to normal individuals, by dynamic recording of cardiac function as well as simultaneous monitoring of regional perfusion of the muscle. These findings demonstrate that cardiac response even to submaximal exercise is attenuated significantly in diabetic individuals. Simultaneously, there is an attempt to maintain regional microvascular perfusion by a compensatory augmentation of regional flow to the exercising muscle. Despite this compensation, there is greater desaturation of the supply reaching the working muscle in diabetics compared with normal individuals. Furthermore, the reduction in cardiac reserve as well as regional oxygen delivery during exercise, were found to be related to the severity of diabetes.

Reduced exercise capacity in diabetics has been reported in subjects with non-insulin dependent diabetes mellitus (NIDDM) without coronary artery disease^8^ and a correlation between left ventricular dysfunction and impaired exercise capacity has been demonstrated in diabetics in several studies.^9^ Our study confirms that the SV reserve is the key parameter that is compromised during exercise in asymptomatic diabetic individuals. This is evident even with the mild exercise equivalent to a 6-minute walk test when measured dynamically in ambulatory subjects who are not in failure. The association of poor glycaemic control with worse cardiac and peripheral dysfunction in this study is consistent with a previous large study in asymptomatic type 2 diabetics.^10^ Other studies have shown that A_1c_ has an inverse correlation with maximum oxygen uptake,^11^ work capacity^12^ and exercise duration.^13^

Perturbation of cardiac function independent of coronary artery disease and BP in diabetes may result from an interplay of a wide variety of progressively dysfunctional autoregulatory mechanisms. Diastolic abnormalities like delayed onset of ventricular relaxation,^14^ and systolic function abnormalities of the ventricular muscular pump characterised by abnormal systolic time intervals and by increased brain natriuretic peptide (BNP) levels, as observed in this study, precede reduced haemodynamic pump performance with severe systolic and diastolic abnormalities. Limitations on cardiac reserve and cardiac contractile function thus restrict the supply from meeting the demand imposed by exercise. In addition, microcirculatory deficits coupled with altered energetics limit the ability to meet the demand in exercising skeletal muscle in diabetics.^15^ ^16^ Endothelial dysfunction and functional alterations of the microcirculation, on the other hand, precede morphological changes and contribute to an increased vascular permeability^17^ and an impaired endothelial vasodilatory response. There are several reports that insulin also increases sympathetic nervous system activity, which can determine the nutritive flow distribution.^18^ Diabetes with associated insulin resistance and autonomic dysfunction could lead to further exacerbation of the maldistribution locally to fulfil the nutritive flow required to meet the metabolic demand during exercise. These changes, acting in conjunction, induce dysfunction with resultant reductions in flow and O_2_ delivery at rest and during exercise.^19^ A perturbation of intramuscular homeostasis in response to exercise challenge was also observed in this study, which has the potential to contribute to premature muscular fatigue and progress to reduced exercise tolerance in diabetic individuals.

During the recovery period, a shift from carbohydrate to fat as a fuel substrate occurs to prevent further depletion of muscle glycogen stores, leading to oxygen consumption remaining elevated above resting levels after exercise, called oxygen debt, because the oxygen cost of fat catabolism is greater than that of carbohydrate catabolism.^20^ ^21^ In order to meet this requirement, a marked increase in stroke volume during early recovery with overshoot appears to be facilitated by both an immediate afterload reduction and a relatively slow decrease in cardiac sympathetic stimulation. Accumulation of a large oxygen debt during exercise has been reported in heart failure patients.^22^ In this study, overshoot of cardiac output was observed during recovery, though lower in diabetics and so was the mixed venous SO_2_, suggesting more severe muscle underperfusion and greater O_2_-deficit and regional ischaemia in diabetic individuals. A possible mechanism could be a persistence of sympathetic activity, which serves to increase vascular resistance in non-working tissues during exercise, which the local metabolic factors and endothelium-derived substances such as nitric oxide and prostaglandins are unable to over-ride, as they are deficient in the diabetic state. Increased renin-angiotensin-endothelin system activity may also contribute, at least in part.

Mediators of the changes underlying impaired exercise response in patients with type 2 diabetes are multiple. Limited exercise tolerance has been associated with poor glycaemic control.^8^ ^10^ ^11^ ^12^ ^23^ Secondary to this, glycosylation leading to the formation of AGE (advanced glycation endproduct), initiate a proinflammatory and procoagulant state and may impair the function of a number of proteins leading to cardiac, vascular or endothelial dysfunction.^24^ ^25^ We have found that diabetes-induced myocardial dysfunction in a diabetic hypertensive rat model was reversed by reducing the AGE burden consequent to treatment with AGE-breaker.^26^

Previous reports of ICG in diabetics have been limited and restricted to measurements at rest. One ICG study in diabetic individuals revealed that thoracic fluid content increased despite improvement in contractile indices after treatment with rosiglitazone.^27^ In another study, no difference was detected in cardiac index at rest between healthy individuals and newly diagnosed diabetics,^28^ indicating that possibly exercise provocation would be necessary to bring out the subtle differences in the haemodynamics. Insights on the control of muscle blood flow and oxygen consumption during exercise have, in the past, relied on measurements of bulk flow in large conduit arteries to the exercising limbs precluding analysis of local perfusion and oxygenation mismatch. However, comparing changes during rest, dynamic exercise and recovery after exercise with a combination of NIRS, laser Doppler and impedance cardiography to expose subclinical differences in diabetic individuals has not been attempted previously to the best of our knowledge. Repeatability of the measurements showed reasonable consistency between measurements made on both O2C and ICG parameters.

The potential limitations of our study are the mixed diabetic population studied, which could have incipient complications like neuropathy, peripheral vascular disease and coronary artery disease, which could be influencing the study parameters. For diabetic subjects without any signs of heart failure, reduction in exercise capacity secondary to reduction in cardiac output may be evident only during maximal exercise and could be of prognostic significance. We also do not have tissue-Doppler data to correlate limitation in increasing stroke volume to diastolic dysfunction.

## Conclusion

In summary, this cross-sectional study in a diabetic population showed that by combining the assessment of dynamic cardiac response during exercise with peripheral flow distribution and nutritive perfusion, subclinical differences in haemodynamic response were detected in diabetic individuals, hitherto not reported to the best of our knowledge. Longitudinal follow-up of these patients and a comparison of dynamic cardiac response and that of peripheral flow and nutritive distribution, as described, with the traditional measures of exercise capacity, which have been studied longitudinally over time and shown to have prognostic significance, would provide an objective and sensitive measure of cardiac and microvascular correlates of the composite haemodynamic function and indicate the suitability of such diagnostic measures for early detection of a population at risk of progressing to heart failure.^5^
